# Animal Evidence for Synergistic Induction of Hepatic Injury by Dietary Fat and Alcohol Consumption and Its Potential Mechanisms

**DOI:** 10.3390/jpm11040287

**Published:** 2021-04-08

**Authors:** Hyeong-Geug Kim, Jing-Hua Wang, Hyo-Seon Kim, Jin-Seok Lee, Hwi-Jin Im, Sung-Bae Lee, Dong-soo Lee, Gang-Min Hur, Chang-Gue Son

**Affiliations:** 1 Institute of Bioscience & Integrative Medicine, Daejeon University, 75, Daedeok-daero 176, Seo-gu, Daejeon 35235, Korea; winakim@hanmail.net (H.-G.K.); ewccwang@gmail.com (J.-H.W.); khs910707@hanmail.net (H.-S.K.); neptune@dju.kr (J.-S.L.); lastdohee@gmail.com (H.-J.I.); sky161300@naver.com (S.-B.L.); 2 Liver and Immunology Research Center, Daejeon Korean Medicine Hospital, 75, Daedeok-daero 176, Seo-gu, Daejeon 35235, Korea; 3 Department of Internal Medicine, Daejeon St. Mary’s Hospital, College of Medicine, The Catholic University of Korea, 64, Daeheung-ro, Jung-gu, Daejeon 34943, Korea; endoscope@hanmail.net; 4 Department of Pharmacology, Research Institute for Medical Science, College of Medicine, Chungnam National University, Daejeon 301131, Korea; gmhur@cnu.ac.kr

**Keywords:** steatohepatitis, metabolic associated fatty liver disease, alcoholic liver diseases, endoplasmic reticulum stress, mitochondria, NAFLD

## Abstract

In contrast to nonalcoholic fatty liver disease (NAFLD), metabolic-associated fatty liver disease (MAFLD) as an innovative definition can coexist with significant alcohol consumption. Massive clinical observations have indicated that high-fat/-calorie diet induced metabolic dysfunction along with alcohol intake deteriorates steatotic liver injury. To explore the potential mechanisms of fatty diet together with alcohol-induced steatohepatitis, we adopted a rat model by comparing a half-dose combination of fat diet (20%) and alcohol (10%) with their corresponding double dose of 40% fat diet and 20% alcohol for 8 weeks. The notable alterations in histopathology, acceleration in the oxidation parameters (ROS, NO and lipid peroxidation) and serum transaminase levels were shown in the concomitant group. Concomitant use of a high-fat diet and alcohol provoked hepatic endoplasmic reticulum stress, but did not activate mitochondria-mediated apoptosis parameters compared to F. In contrast, the notable activation of caspase-12 and nuclear translocation of CCAAT/enhancer-binding protein (C/EBP) homologous protein (CHOP) were observed only in the combined treatment group. The concomitant dietary fat intake and alcohol consumption lead to liver injury initially and later to steatohepatitis by the overdose of fat or alcohol, and in which the CHOP and caspase-12 might be involved in synergistic acceleration of steatohepatitis through a mitochondria-independent manner.

## 1. Introduction

The decreased carrier of hepatic viruses and an increase in the population suffering from obesity lead to high-fat diets and alcohol abuse becoming major causes of liver diseases, especially in industrialized countries [1,2]. The public vaccination against hepatitis B virus (HBV) rapidly reduced chronic HBV carriers from 9.8% to 3.1% over recent 20 years in China [3], and recent antiviral therapies achieved the sustained virological response by 91% of patients with Hepatitis C virus (HCV) in Japan [4]. Instead, as we know, metabolic dysfunctions and alcohol abuse lead to 13% and 9% of chronic liver diseases, respectively, in Western European countries [5].

In clinics, steatohepatitis has been simply categorized into two distinct types depending on the absence of significant alcohol drinking since 1980: nonalcoholic steatohepatitis (NASH) and alcoholic steatotic hepatitis (ASH) [6]. NASH and ASH have a different etiology depending on the clinical diagnostic criteria, such as the amount of alcohol consumption (generally 20 g/day for women and 30 g/day for men) [7]. However, their pathological features and histopathological characteristics are too similar, almost indistinguishable in terms of excessive fat accumulation and cellular damage [8]. They are both provoked by a complex process involving the imbalance between lipogenesis and lipolysis, as well as an increase of the imported fat to the liver, followed by harmful events, including an inflammatory response [9,10]. Furthermore, clinically most patients with alcoholic liver disease also commonly experience abnormal diet habits, such as oversupplying high calories/fat containing diets [11]. Despite the ongoing debate [12,13], recently scientists and clinicians realized that the oversimplified segregation of steatohepatitis into NASH and ASH has obvious limitations, such as the exclusion of alcohol intake, “one-size-fits-all” approach. Thus, metabolic-associated fatty liver disease (MAFLD) is increasingly recognized as the more appropriate nomenclature than non-alcoholic fatty liver disease (NAFLD) owing to better interpretation of heterogeneous pathogenesis, including co-existence with alcoholic consumption and other inducers [14].

Actually, the synergistic risky effect of high fat diet (HFD) and alcohol consumption was well evidenced by an epidemiologic study, in which individuals with both chronic alcohol consumption and excessive nutrition had a higher risk of liver injury than subjects with heavy alcohol or high-fat diets separately [15]. Combination of alcohol and chronic alcohol consumption plus excessive nutrition supply accelerated liver injury and the development of fibrosis > two or three-fold in other clinical observations [16,17]. Moreover, one recent clinical meta-analysis showed that MAFLD patients with alcohol consumption have more severe liver injury than those without [18]. In animal models, intermittent alcohol drinking and HFD intake led to more severe liver injury than either alcohol or HFD alone [19]. In addition, several other animal studies also showed the increased susceptibility to hepatic injury by simultaneous intake of a HFD and alcohol [20,21,22]. However, these studies used the animal designs resulting in predictable outcomes, like two risk factors (alcohol plus fat diet/over calorie) versus each factor with the same quantity. Their synergistic effects on liver injury were explained by alteration of innate immunity, enhancement of pro-fibrotic signaling, increase of de novo lipogenesis (DNL) or preceding mitochondrial-dependent apoptosis signals [19,22,23], however, the accurate pathological mechanisms have not been revealed clearly.

To verify the pathogenic effects comprehensively by concomitant use of fat diet and alcohol, we herein adapted a rat model, comparing each factor (40% fat diet or 20% alcohol) versus co-consumption of their half-quantity (20% fat diet plus 10% alcohol), and then we investigated the underlying mechanisms of the synergistic mode of hepatic injury.

## 2. Materials and Methods

### 2.1. Animals and Experimental Design

All animals were treated in accordance with the National Institutes of Health Guide for Care and Use of Laboratory Animals and the current study was approved by the Institutional Animal Care and Use Committee of Daejeon University (DJUARB-2015010). Sprague-Dawley rats (specific pathogen-free, male 210–230 g, 8 weeks of age) were purchased (Dae-han biolink, Chung-buk, Korea) and freely accessed tap water and a standard diet. After 7-day acclimatization, rats were randomly divided into six groups (*n* = 6 for each group): control (distilled water; DW with a normal chow diet), fat diet 20 (DW with 20% fat diet), fat diet 40 (DW with 40% fat diet), ethanol 10 (10% ethanol/kg/day with a normal chow diet), ethanol 20 (20% ethanol/kg/day with standard normal chow diet) and fat diet 20 plus ethanol 10 (20% fat diet with 10% ethanol/kg/day) groups. Rats were orally administered with DW or ethanol (10 mL/kg daily) using gastric gavages, and freely accessed the normal and fat diet (20% or 40%, Research diet, Inc. manufactured ID #D14052401 for 20% fat diet and #D14052402 for 40% fat diet, Table S1) for 8 weeks.

Total body weight was recorded weekly and whole blood was collected from the abdominal common artery under ether anesthesia on the final day under 9 h of fasting. The liver and abdominal fat tissues were removed and weighed, and then liver tissues were fixed in 10% neutral formalin, Bonin’s solution or stored in RNAlater (Ambion, Austin, TX, USA) or at −70 °C for further examination.

### 2.2. Serum Biochemistry Analysis

After 1 h blood clotting in room temperature, serum was separated from collected blood using centrifugation with 3000× *g* for 15 min at 4 °C. Serum levels of aspartate transaminase (AST), and alanine transaminase (ALT), and alkaline phosphatase (ALP), triglycerides (TG), total cholesterol (TC), low-density lipoprotein (LDL), and glucose were analyzed using a Chemistry Auto Analyzer (Chiron, Emeryville, CA, USA). Besides, serum level of free fatty acid (FFA) was determined by automatic biochemistry analysis system (AU480, Beckman coulter, Brea, CA, USA).

### 2.3. Lipid Contents Determinations

Hepatic tissue levels of lipid contents such as TG and TC were measured by previous methods [1,3,24,25]. Briefly, to determine hepatic TG levels, 100 mg of each rat liver tissue samples were homogenized in phosphate-buffered saline (PBS, 10 mM, pH 7.2) and then centrifuged. The supernatant was mixed with 10% Triton-100 in PBS for measuring TG using a commercial kit (Asan Pharmaceutical Co., Ltd., Seoul, Korea). For measuring hepatic TC, 50 mg of liver tissue was homogenized in 1 mL of chloroform-methanol mixture (2:1, *v*/*v*). After centrifugation at 10,000× *g* for 15 min, the supernatant was dried at room temperature. Then, 200 µL of 10% Triton-100 in PBS was added to each sample. Next, TC levels were measured by routine methods using the appropriate commercial kit (Asan Pharmaceutical Co., Ltd., Seoul, Korea).

### 2.4. Histopathological Analysis

Liver tissue was fixed in 10% neutral formalin solution for analyzing the histopathological analysis. For hematoxylin and eosin (H&E) staining and immunohistochemistry (IHC) against 4-hydroxy-2-nonenal (4-HNE), terminal deoxynucleotidyl transferase dUTP nick end labeling (TUNEL), myeloperoxidase (MPO) and F4/80, paraffin-embedded sections (5-μm of thickness) were used, while oil-red O staining was performed using frozen liver tissue sections (8-μm of thickness). IHC analyses were followed by manufactures’ manuals and signals were detected by 3,3′-diaminobenzidine (DAB) or 3-amino-9-ethylcarbazole (AEC) substrate. The quantitative analyses of inflamed cell infiltration, lipid accumulation areas were performed by previous studies with slight modification [26,27,28]. The intensity or counts of positive signals were quantified from the randomly selected at least five fields of each sample using Image J software (version 1.52v; National Institutes of Health, Bethesda, MD, USA).

### 2.5. Immunohistofluorescence (IHF) Analysis

Frozen liver sections (8-μm) were fixed in acetone (−20 °C for 20 min) and washed with tap water in a running type, then slides were incubated a blocking buffer (5% normal serum in 0.3% Triton™ X-100 in a 10 mM PBS) for 40 min. Then, mouse-anti-CHOP (CCAAT-enhancer-binding protein homologous protein) antibody (diluted in 1:100, #2895, Cell Signaling) with the antibody dilution buffer (1% BSA with 0.3% Triton™ X-100 in 10 mM PBS) was applied and incubated overnight. After incubation, the rabbit-α-tubulin antibody (diluted 1:1000, #sc-5546, Santa Cruz, Dallas, TX, USA) further incubated for overnight at RT. After washing the primary antibodies, secondary goat anti-mouse or rabbit IgG (H+L) secondary antibodies (Goat-anti-mouse Alexa Fluor^®^ 488 conjugate and Goat-anti-rabbit-Alexa Fluor^®^ 594 conjugate) was applied. The cell-based immunofluorescence analyses were conducted according to the manufacture’ protocol against CHOP. All histopathological features were examined under microscopy circumstance (200× or 400× magnification, IX71; Olympus, Tokyo, Japan).

### 2.6. Caspase-3/7 and Poly (ADP-Ribose) Polymerase (PARP) Activity Analysis

Caspase-3/7 activity in the hepatic tissue was determined using the commercial product (PerkinElmer, Waltham, MA, USA) according to the manufacturer’s protocol and the fluorescence intensity was determined using a Perkin-Elmer Victor 3 instrument. PARP activity in the hepatic tissue homogenates was assessed using a colorimetric assay kit (Trevigen Inc., Gaithersburg, MD, USA).

### 2.7. Western Blot Analysis

Western blotting analyses were conducted for evaluation of apoptosis, ER stress and lipid metabolism-related molecules. Whole fractions (cytosolic, nuclear, or mitochondrial faction) in the liver tissue were homogenized using RIPA buffer or commercial kit (NE-PER™ Nuclear and Cytoplasmic Extraction Reagents and Mitochondria Isolation Kit for Tissue Cat. No. #89801, Thermo Fisher Scientific, Waltham, MA, USA). A total of 40 μg of each protein was separated by 10% polyacrylamide gel electrophoresis and transferred to polyvinylidene fluoride (PVDF) membranes. After blocking in 5% skim milk, the membranes were probed overnight at 4 °C with primary antibodies (the uses of primary antibodies for western blotting were based on the manufacturer’s instructions, Table S2). The membranes were washed three times and incubated for 2 h with HRP-conjugated anti-rabbit antibody. Western blots were visualized using an enhanced chemiluminescence kit.

### 2.8. mRNA Expression Analysis Using qPCR

The mRNA expression levels in liver tissue were analyzed by the performance of quantitative polymerase chain reaction (qPCR). Total RNA was isolated from liver tissue samples using TRIzol reagent (Molecular Research Center, Cincinnati, OH, USA). cDNA was then synthesized from total RNA (2 μg) in a 20 μL reaction using a high-capacity cDNA reverse transcription kit (Ambion^®^). The qPCR was performed using SYBR Green PCR Master Mix (Applied Biosystems, Foster City, CA, USA), and PCR amplification was performed using a standard protocol with the IQ5 PCR Thermal Cycler (Bio-Rad, Hercules, CA, USA). For data analysis, the gene expression levels were compared with those of β-actin as a reference gene. The information regarding each primer sequence was provided in the Table S3.

### 2.9. Statistical Analysis

All data are expressed as the mean ± standard deviation (SD). Statistically significant differences among the groups were analyzed by one-way ANOVA followed by Fisher’s least significant difference (LSD) post-hoc test using the SPSS statistics (17.0 version, Chicago, IL, USA). The difference at *p* < 0.05 was considered statistically significant.

## 3. Results

### 3.1. Synergistic Induction of Hepatosteatosis and Liver Injury

**Figure 1:**
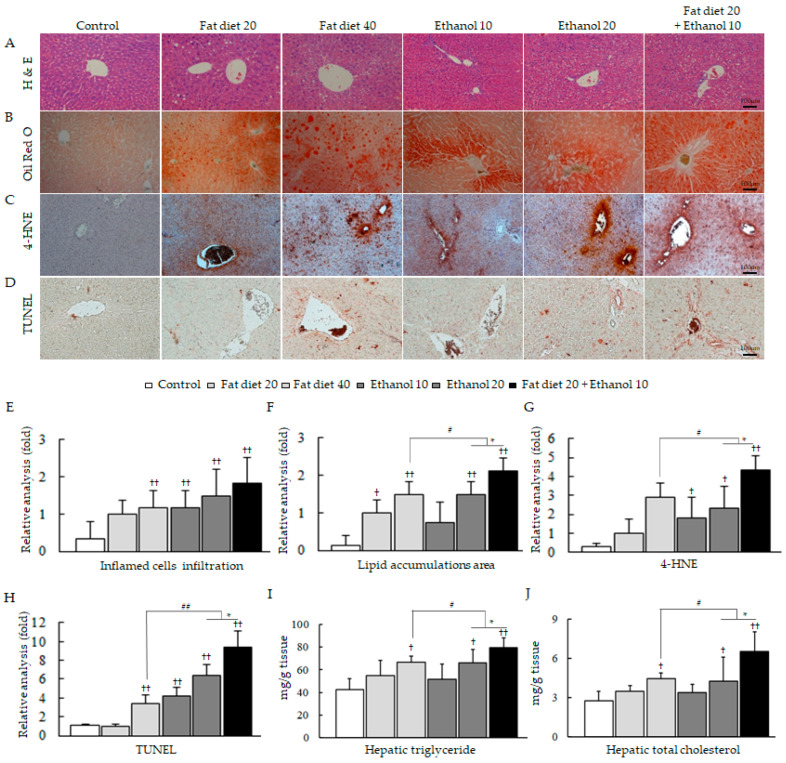
Histopathological analyses and hepatic lipid profiles. Representative photomicrographs of liver tissues for hematoxylin and eosin (H&E) staining (**A**), oil red O staining (**B**), immunohistochemistry (IHC) for 4-hydroxy-2-nonenal (4-HNE) (**C**) and terminal deoxynucleotidyl transferase dUTP nick end labeling (TUNEL) (**D**) and their quantitative analyses for inflammatory cell infiltrations (**E**), lipid droplets areas (**F**), intensity or cell count of positive cells (**G**,**H**), fold change comparing to fat diet 20 group are presented. Triglyceride (**I**) and total cholesterol (**J**) levels in hepatic tissue. Data are expressed as the mean ± SD (*n* = 6). ^†^ *p* < 0.05 and ^††^ *p* < 0.01 compared with the control group; ^#^ *p* < 0.05 and ^##^ *p* < 0.01 compared with the fat diet 40 group; * *p* < 0.05 compared with the ethanol 20 group. The photograph was obtained using a light or fluorescence microscope (×200).

The body weights and visceral fat weights were significantly increased in the fat diet 40 and fat diet 20 groups followed by the combined treatment group (fat diet 20 + ethanol 10) and the ethanol 20 and ethanol 10 group (Table 1), whereas the liver tissue weights and hepatic lipid contents (TC and TG) were significantly increased in the combined treatment group compared with the high-fat diet groups or alcohol consumption groups (*p* < 0.05, Table 1, Figure 1I,J). Moreover, the serum levels of total TC, LDL, TG, glucose and free fatty acid were significantly higher in the combined treatment group compared to other groups (*p* < 0.05 or 0.01, Table 1).

Abnormal serum hepatic biochemical parameters (AST, ALT and ALP) were observed only in the combined treatment group (Table 1). The above results conformed to histopathological findings by H&E, oil red O and 4-HNE staining and TUNEL assay (Figure 1A–H).

The average amounts of weekly diet intake were not markedly different among all groups through whole experiment periods (Figure S1A–D). The body weight gain was high in fat diet 40 group but low in ethanol 20 group, which was significantly changed with the combined treatment group (*p* < 0.05 or 0.01). The liver findings by naked eye also were in accordance with above alterations (Figure S1A–D).

### 3.2. Exacerbation of Oxidative Stress and Hepatic Inflammation

**Figure 2:**
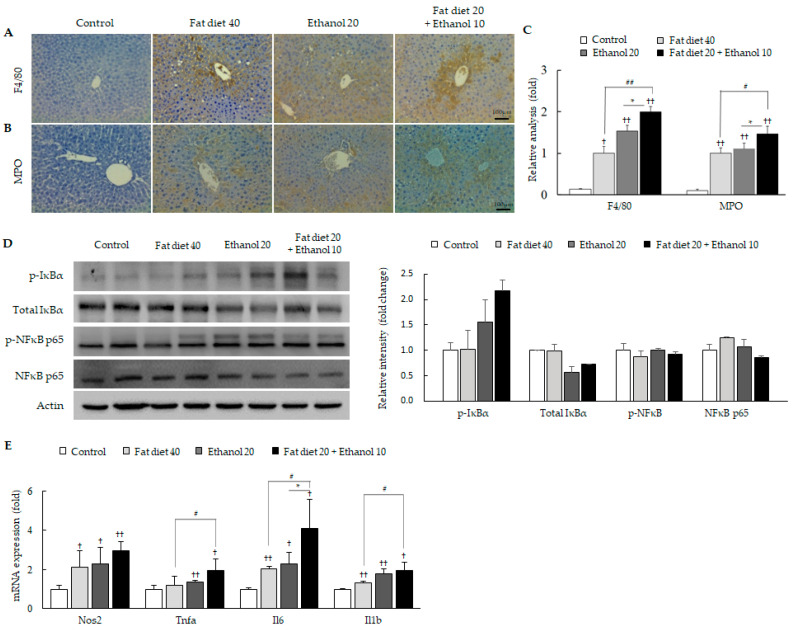
Inflammation phenotypes of hepatic tissues. Immunohistochemistry analysis against F4/80 (**A**) and MPO (**B**) and their quantitative analyses intensity of positive cells (**C**), fold change comparing to fat diet 40 group are presented. Western blot and relative intensity analysis of inflammatory regulator molecules (**D**) and mRNA expression levels of pro-inflammatory cytokines (**E**) are presented. Data are expressed as the mean ± SD (*n* = 6). ^†^ *p* < 0.05 and ^††^ *p* < 0.01 compared with the control group; ^#^ *p* < 0.05 and ^##^ *p* < 0.01 compared with the fat diet 40 group; * *p* < 0.05 compared with the ethanol 20 group. The photograph was obtained using a light or fluorescence microscope (×200).

IHC staining for 4-HNE (*p* < 0.05, Figure 1C) and oxidative stress parameters including the hepatic level of malondialdehyde (MDA), reactive oxygen species (ROS), nitric oxide (NO) and protein carbonyl (*p* < 0.05, Figure S2A–D) showed that the combined treatment group had significant alterations compared with only high-fat diet groups or only alcohol consumption groups. Inflammatory cell infiltration and activation was also distinguished in the combined treatment group compared with their counterparts, which was evidenced by H&E staining and F4/80 and MPO IHC (Figure 1A and Figure 2A–C).

The combined treatment also induced the notable activation of the pro-inflammatory molecules, including IκBα and NF-κB (p65) in western blotting (Figure 2D) and significant up-regulation of three pro-inflammatory cytokines (tumor necrosis factor-alpha; Tnfa, interleukin-6; Il6 and Il1b) in genes expression compared with their counterparts, respectively (*p* < 0.05, Figure 2E).

### 3.3. Synergistic Induction of Endoplasmic Reticulum (ER) Stress

**Figure 3:**
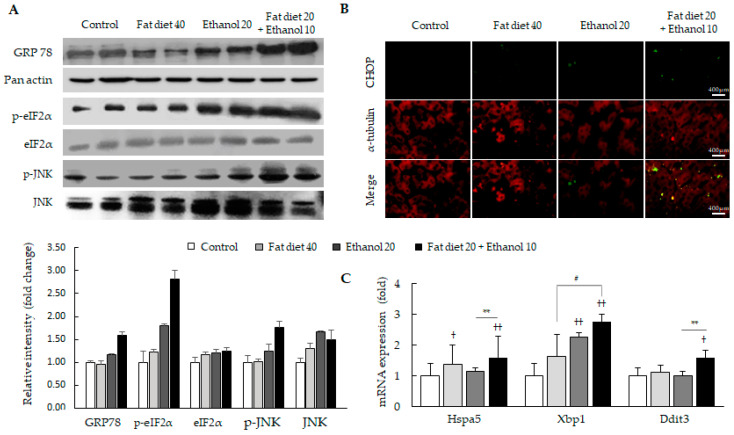
Alterations in hepatic ER stress-related molecules. Western blot and relative intensity analysis of hepatic ER stress-related molecules (**A**), IHC for CCAAT-enhancer-binding protein homologous protein (CHOP) activation (**B**) and mRNA expression of ER stress-related core genes (**C**) are presented. Data are expressed as the mean ± SD (*n* = 6). ^†^ *p* < 0.05 and ^††^ *p* < 0.01 compared with the control group; ^#^ *p* < 0.05 compared with the fat diet 40 group. ** *p* < 0.01 compared with the ethanol 20 group. The fluorescence signals were detected using a fluorescent microscope (×400).

The ER stress parameters, glucose-regulated protein 78 (GRP78), and eukaryotic initiation factor 2α (eIF2α) and c-Jun N-terminal kinases (JNK), were notably enhanced in the combined treatment group, but slightly in ethanol 20 group (Figure 3A). Nuclear translocation of CHOP was also drastically augmented in the combined treatment group compared with the other groups (Figure 3B). These results were consistent with the mRNA expressions of ER-stress associated genes (heat shock 70 kDa protein 5; Hspa5, X-box binding protein 1; Xbp1, and DNA-damage-inducible transcript 3; Ddit3), respectively (*p* < 0.05 or 0.01, Figure 3C).

### 3.4. Induction of Mitochondria-Independent Apoptosis

**Figure 4:**
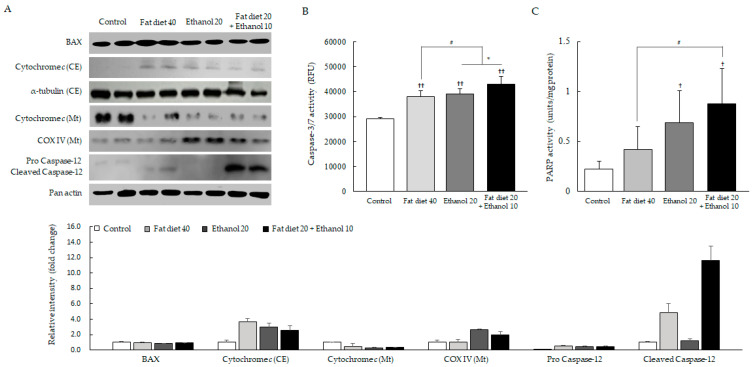
Alterations of apoptosis-related molecules in hepatic tissue. Western blot analysis and relative intensity analysis of apoptosis-related molecules (**A**), caspase 3/7 activity (**B**) and PARP activity (**C**) in hepatic tissue are presented. Data are expressed as the mean ± SD (*n* = 6). ^†^ *p* < 0.05 and ^††^ *p* < 0.01 compared with the control group; ^#^ *p* < 0.05 compared with the fat diet 40 group. * *p* < 0.05 compared with the ethanol 20 group.

Notable difference was not observed in two key parameters of mitochondrial apoptosis (cytochrome c and B-cell lymphoma 2-associated X protein; BAX) between the combined treatment group and their counterpart groups (Figure 4A). In contrast, caspase-12 protein, which mainly involved mitochondrial-independent apoptosis molecule, was considerably activated only in the combined treatment group compared with the high-fat diet group or alcohol consumption group (Figure 4A). Additionally, two key apoptosis related enzymes, caspase-3/7 and PARP activities were significantly higher in the combined group than those of counterparts (fat diet 40% or ethanol 20%), respectively (*p* < 0.05, Figure 4B,C).

### 3.5. Synergistic Alterations of Lipid Homeostasis

**Figure 5:**
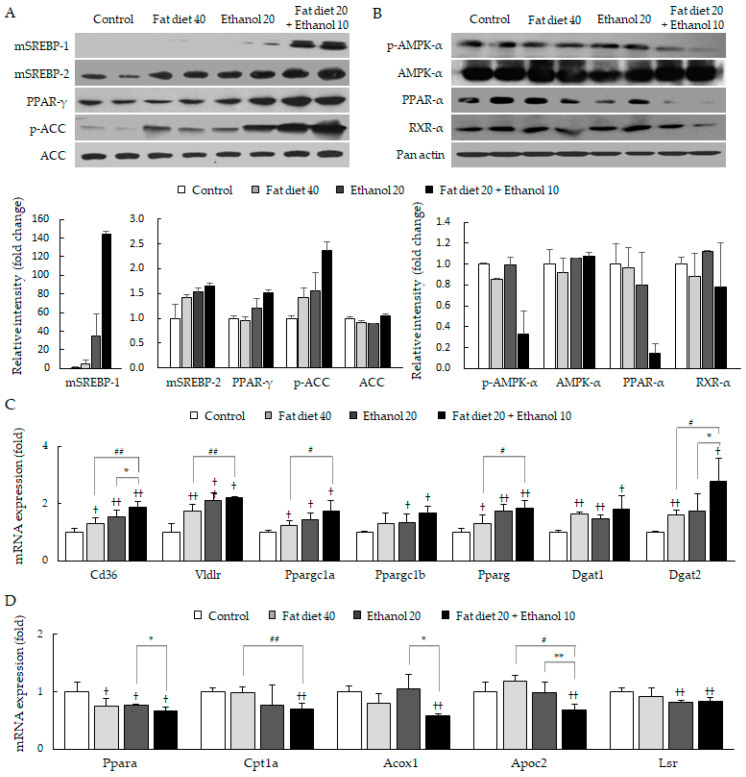
Impairments in lipid homeostasis in hepatic tissues. Western blot and relative intensity analysis for core molecules of lipogenesis (**A**) and lipolysis (**B**), and mRNA expression analyses for core genes of lipogenesis (**C**) and lipolysis (**D**) in hepatic tissues are presented. Data are expressed as the mean ± SD (*n* = 6). ^†^ *p* < 0.05 and ^††^ *p* < 0.01 compared with the control group; ^#^ *p* < 0.05 and ^##^ *p* < 0.01 compared with the fat diet 40 group; * *p* < 0.05 and ** *p* < 0.01 compared with the ethanol 20 group.

Compared with the fat diet 40 group or ethanol 20 group, the combined treatment group notably elevated hepatic protein activities of lipogenesis-related molecules including sterol regulatory element-binding protein (SREBP)-1 and -2, peroxisome proliferator-activated receptor (PPAR)-γ, and acetyl-CoA carboxylase (ACC), respectively (Figure 5A). Meanwhile, the protein levels of lipolytic molecules including AMP-activated protein kinase (AMPK)-α, PPAR-α and retinoid X receptor (RXR)-α were markedly decreased in the combined treatment group (Figure 5B).

The combined treatment also significantly up-regulated the mRNA expressions of genes involved in lipid uptake, de novo lipogenesis and lipid droplet formation (*p* < 0.05 or 0.01, Figure 5C). Conversely, the mRNA expressions of lipolytic core molecules were down-regulated in the combined treatment group compared with their counterpart groups (*p* < 0.05 or 0.01, Figure 5D).

## 4. Discussion

As critical inducers of hepatic steatosis, both excess fat or calorie and chronic ethanol consumption are deeply associated with the recent burden of metabolic syndromes [29,30]. Our results well evidenced that concomitant intake of dietary fat and alcohol (even in half quantity) is a higher risky condition in the development of fatty liver, hyperlipidemia and hyperglycemia, compared to the high dose of the single factor (high-fat diet or alcohol) alone (Figure 1B,F,I,J, Table 1). Our data also revealed that the systematic FFA has been synergistically elevated in the concomitant half dose of dietary fat and alcohol consumption group. As we know, hepatic steatosis is initiated by the imbalance of free fatty acid (FFA) metabolism, between oxidation of FFAs to generate adenosine triphosphate (ATP) and production of TG, respectively [31]. The overproduction of FFAs inside hepatocytes leads to activation of lipogenic molecules including SREBP-1 and PPAR-γ, whereas deters lipolytic molecules such as PPAR-α and AMPK-α, respectively [32]. The excessive consumption of fat raises lipogenesis-dominant circumstance, whereas alcohol abuse inhibits the lipolysis pathway, which can lead to accelerating the lipogenesis signaling [33]. Our results represented well the remarkable alterations of molecular parameters of lipid metabolism in concomitant usage of fatty food and alcohol (even with a half-dose) compared to their counterparts alone with a full dose, respectively (Figure 5A–D).

HFD indirectly interfere liver function through disruption of energy homeostasis regulation [34], whereas alcohol directly induce liver injury via intermediate metabolite, acetaldehyde [35]. As we expected, concomitant consumption of fat diet and alcohol easily induced hepatic damage, evidenced by elevated levels of serum PP, ALT and ALP (Table 1). In particular, ALP level was significantly increased by the combined group comparing to high-fat diet or alcohol alone, which is a typical enzyme indicating cholestasis disorder in liver [36]. However, treatment of HFD alone induced mild liver injury in the present study as compared to other HFD study [37]. It might be due to the uncertain factors, such as environment and individual difference. Oxidative stress is well known as a common contributor in both fat- and alcohol-caused hepatic injury [38,39], and then the combined treatment with fat diet plus ethanol significantly augmented oxidative stress condition comparing to their double dose of counterparts (Figure 1C,G, Figure S2A–H). As anticipated, these oxidative alterations were linked to the enhanced hepatic inflammation, evidenced by histopathological findings from H&E staining and IHC against MPO and F4/80 (Figure 1A and Figure 2A–C). Further, these findings were well supported by analyzing the representative molecule for inflammatory protein (NF-κB) and gene expression of pro-inflammatory cytokines including Nos2, Tnfα, Il-6 and Il1β, respectively (Figure 2D,E). MPO and F4/80 are markers of neutrophil activation and M1 phase of macrophage in the liver tissues, which mainly contribute to the production of ROS and inflammatory circumstances under pathogenic conditions [40].

On the other hand, ER stress is the main event in the progress of hepatic apoptosis in either fat diet or ethanol abuse-induced steatotic liver injury [41]. Excessive exposure of fat diet or alcohol to the liver tissue causes ER stress via unfolded protein response in the lumen of the ER, which activates GRP78 [42]. GRP78 positively regulates initiation mediators of ER stress, including protein kinase RNA-like endoplasmic reticulum kinase (PERK) and inositol requiring enzyme 1α (IRE1α) [43,44]. In our results, the concomitant intake of dietary fat and alcohol consumption notably activated the GRP78 including eIF2α and JNK, the two main downstream molecules of PERK and IRE1α, respectively (Figure 3A). This condition remarkably activated the hepatic apoptosis induced by ER stress as shown in TUNNEL assay and immunostaining of CHOP comparing to both high-fat and ethanol group (Figure 1D,H and Figure 3B). CHOP is well noticed as a determinant marker of ER stress-induced apoptosis as a mitochondria-independent pathway [45]. These pathological conditions were well reenacted by mRNA expression levels of genes involved in ER stress, including Hspa5, Xbp1 and Ddit3 (Figure 3C).

Under ER stress condition, the ROS can be generated in both ER and mitochondria [46]. ER stress induces mitochondrial apoptosis via activation of pro-apoptotic molecules such as BAX, followed by the release of cytochrome c and finally activation of caspase-3/7 in steatohepatitis [47]. In this process, JNK plays a key role as a pro-apoptotic mediator [48]. In our study, the concomitant consumption of fat diet and ethanol remarkably induced the activation of JNK and augmented the activities of caspase-3/7 and PARP comparing to their counterparts (Figure 3A and Figure 4B,C). PARP is a potent marker of apoptosis-induced DNA fragmentation and co-activator of key pro-inflammatory transcription factors [49,50]. However, interestingly the release of cytochrome c and BAX activation, two vital molecules of mitochondria-dependent apoptosis, did not differ among the three groups (fat diet 40, ethanol 20 and combined treatment group) (Figure 4A). Those results proposed another pathway for the induction of hepatic injury beside mitochondria-dependent apoptosis under the condition of concomitant consumption of fat diet and ethanol. Based on the report for the novel pathway of the caspase-12 in a mitochondria-independent apoptosis [51], we examined the levels of the caspase-12 and found drastic activation only in the combined treatment group (Figure 4A and Figure S3). This mitochondria-independent caspase-12 pathway also occurs via the partial contribution of CHOP under ER stress [52]. Our results also evidenced the notable the nuclear translocation of CHOP in the combined treatments group of high fat and ethanol (Figure 3B).

Above results indicated that the concomitant intake of dietary fat and alcohol can lead to liver injury prior to induction of steatohepatitis by the overdose of fat or alcohol, and then CHOP and caspase-12 are involved in this process. Previous clinical studies claimed the vulnerable hepatic injury by the concurrence of obesity and alcohol consumption [53]. The variety of factors such as gender, genetics and/or lifestyles have complexly interacted in hepatic injury, thus give the difficulties to study for the mechanisms in human. Based on our data, we confirmed the clinical finding that concomitant intake condition of dietary fat and alcohol accelerates hepatic steatosis and liver injury even under moderate load [54]. In addition, the underlying mechanisms may involve the early augmentation of ER stress resulting in caspase-12 and CHOP activation. Alcohol should be metabolized prior to other nutritional components in the liver. Nevertheless, we still cannot identify the uniqueness of the mechanisms for the above outcome. So, Caspase 12 and/or CHOP deficiency animal model would be used in further study. Eventually, these findings will be conducive to offer scientific evidence and guidelines for healthy eating and hepatic protection. Therefore, we herein suggest to avoid not only heavy drinking and habitual HFD eating but also simultaneous intake of alcohol and HFD even in moderate amounts.

## 5. Conclusions

Taken together, we conclude that concomitant condition of dietary fat intake (or obesity) and alcohol consumption triggers the development of steatohepatitis early and its acceleration via mitochondria-independent manner involving the activation of caspase-12 and CHOP. Our findings would contribute to understanding the pathophysiology of MAFLD and approaching its therapeutics.

## Figures and Tables

**Table 1:** Changes of body, organ mass and serum biochemistries.

Contents	Control	Fat Diet 20%	Fat Diet 40%	Ethanol 10%	Ethanol 20%	Fat Diet 20% + Ethanol 10%
Final body weight (g)	462.2 ± 14.8	484.3 ± 9.9	513.2 ± 21.7 ^††^	439.5 ± 21.0	429.0 ± 11.9 ^††^	473.3 ± 26.4 ^#,^**
Liver weights (g)	11.9 ± 0.5	12.8 ± 0.5 ^†^	13.9 ± 0.8 ^††^	12.9 ± 0.7 ^†^	13.8 ± 0.4 ^††^	14.6 ± 0.8 ^††,#,^**
Relative liver weight (%)	2.58 ± 0.01	2.66 ± 0.01	2.69 ± 0.01	2.94 ± 0.01 ^††^	3.21 ± 0.01 ^††^	3.10 ± 0.01 ^††,^^##^
Visceral fat weights (g)	18.8 ± 4.3	27.7 ± 5.0 ^††^	38.2 ± 6.3 ^††^	18.6 ± 3.3	20.2 ± 1.6	26.1 ± 3.9 ^†,^^##,^*
AST (IU/L)	166.5 ± 15.6	162.8 ± 14.3	169.5 ± 15.8	166.3 ± 24.6	189.0 ± 29.1	199.1 ± 47.5 ^†^
ALT (IU/L)	22.8 ± 2.6	26.8 ± 6.0	34.0 ± 11.4	25.8 ± 2.8	33.0 ± 4.0	47.2 ± 26.9 ^††^
ALP (IU/L)	384.8 ±36.4	335.3 ± 41.3	357.8 ± 61.7	363.0 ± 71.2	345.3 ± 23.1	522.0 ± 71.9 ^††,^^##,^**
TC (mg/dL)	58.7 ± 8.0	72.7 ± 7.9 ^†^	73.0 ± 8.4 ^†^	53.0 ± 8.2	64.2 ± 29.8	97.7 ± 16.1 ^††,^^##,^**
LDL (mg/mL)	10.0 ± 0.9	9.5 ± 2.3	9.5 ± 1.2	11.5 ± 2.9	14.0 ± 2.6 ^†^	19.8 ± 3.1 ^††,^^##,^**
TG (mg/dL)	47.3 ± 10.4	70.0 ± 19.4 ^†^	74.7 ± 16.9 ^††^	66.5 ± 18.2	84.7 ± 26.7 ^††^	96.7 ± 13.5 ^†,#^
Glucose (mg/dL)	95.6 ± 11.3	103.7 ± 9.1	104.0 ± 8.0	101.7 ± 11.1	120.3 ± 10.8	184.5 ± 18.4 ^††,^^##,^**
FFA (mEq/L)	0.79 ± 0.18	0.82 ± 0.11	0.86 ± 0.14	1.13 ± 0.12 ^††^	1.45 ± 0.14 ^††^	1.63 ± 0.30 ^††,^^##,^**

Data are expressed as the mean ± SD (*n* = 6, only control group *n* = 8). ^†^ *p* < 0.05 and ^††^ *p* < 0.01 were compared with the control group; ^#^ *p* < 0.05 and ^##^ *p* < 0.01 compared with the fat diet 40; * *p* < 0.05 and ** *p* < 0.01 compared with the ethanol 20 group. AST; aspartate aminotransferase, ALT; alanine aminotransferase, ALP; alkaline phosphatase, TC; total cholesterol, LDL; low-density lipoprotein, TG; triglyceride, FFA; free fatty acid.

## Data Availability

The data that support the findings of this study are available from the corresponding author, [C.-G.S.], upon reasonable request.

## Supplementary Materials

The following are available online at https://www.mdpi.com/article/10.3390/jpm11040287/s1, Figure S1: Changes in body and liver weights and food intake, Figure S2: Hepatic parameters for oxidative stress and antioxidant, Figure S3: Alteration of Capspase-12 activity in HepG2 cells, Table S1: Nutrition compositions of high fat diet, Table S2: Antibodies used in specific application, Table S3: Sequence of the primers used for Q-PCR analysis.

## Footnotes

This manuscript was completed the language editing by the NPG Language Editing Service (ID for NPH61S5S).
