# Supplementary material for: Animal Evidence for Synergistic Induction of Hepatic Injury by Dietary Fat and Alcohol Consumption and Its Potential Mechanisms
Source: J Pers Med. 2021 Apr 8;11(4):287. doi: 10.3390/jpm11040287 (PMC8070044; doi:10.3390/jpm11040287)
Supplement: Supplementary file 1 [file jpm-11-00287-s001.zip › Supplementary tables.docx]

**Supplementary tables**

| **Table S1.** Nutrition compositions of high fat diet | | | | |
| --- | --- | --- | --- | --- |
|  | **Fat diet 20 % (D14052401)** | | **Fat diet 40 % (D14052402)** | |
|  | gm | Kcal | gm | Kcal |
| Protein (%) | 20 | 20 | 23 | 20 |
| Carbohydrate (%) | 61 | 60 | 46 | 40 |
| Fat (%) | 9 | 20 | 20 | 40 |
| Total (%) | - | 100 | - | 100 |
| Kcal/gm | 4.1 | - | 4.6 | - |
| Ingredients | gm | kcal | gm | Kcal |
| Casein, 80 Mesh | 200 | 800 | 200 | 800 |
| L-Cystein | 3 | 12 | 3 | 12 |
| Corn Starch | 321.5 | 1286 | 125.6 | 502 |
| Maltodextrin 10 | 100 | 400 | 100 | 400 |
| Scrose | 172.8 | 691 | 172.8 | 691 |
| Cellulose, BW 200 | 50 | 0 | 50 | 0 |
| Soybean Oil | 25 | 225 | 25 | 225 |
| Lard | 67 | 603 | 154 | 1386 |
| Mineral Mix S10026 | 10 | 0 | 10 | 0 |
| DiCalcium Phosphate | 13 | 0 | 13 | 0 |
| Calcium Carbonate | 5.5 | 0 | 5.5 | 0 |
| Potassium Citrate, 1 H_2_O | 16.5 | 0 | 16.5 | 0 |
| Vitmain Mix V10001 | 10 | 40 | 10 | 40 |
| Choline Bitartrate | 2 | 0 | 2 | 0 |
| FD&C Yellow Dye | 0.05 | 0 | - | - |
| FD&C Blue Dye | - | - | 0.05 | 0 |
| Total | 996.35 | 4057 | 887.45 | 4057 |

| **Table S2.** Antibodies used in specific application | | | | | | |
| --- | --- | --- | --- | --- | --- | --- |
| **Primary Antibody**  **(Catalog #, manufacture, dilution)** | | **Secondary antibody**  **Catalog #, manufacture, dilution)** | | **Application** | | |
| Mouse anti-4 Hydroxynonenal antibody  (#ab46545, Abcam, 1:200) | | UNIVERSAL QUICK KIT Biotinylated PAN-Specific antibody  (#BA-1300, VECTASTATIN, 1:100) | | Immunohistochemistry against 4-HNE | | |
| Rabbit anti-F4/80 antibody  (#ab111101, 1:50) | | UNIVERSAL QUICK KIT Biotinylated PAN-Specific antibody  (#BA-1300, VECTASTATIN, 1:100) | | Immunohistochemistry against MPO | | |
| Rabbit anti-myeloperoxudase antibody  (#ab9535, 1:50) | | UNIVERSAL QUICK KIT Biotinylated PAN-Specific antibody  (#BA-1300, VECTASTATIN, 1:100) | | Immunohistochemistry against F4/80 | | |
| Mouse anti-BAX antibody  (#MA5-14003, Thermo Fisher Scientific, 1:1000) | | Goat anti-Mouse IgG (H+L) Secondary Antibody, HRP conjugate (#31430, Thermo Fisher Scientific, 1:5000) | | Detection of BAX in western blot | | |
| Rabbit anti-cytochrome *c* antibod  (S2050, BD, 1:100) | | Goat anti-Mouse IgG (H+L) Secondary Antibody, HRP conjugate (#31430, Thermo Fisher Scientific, 1:5000) | | Detection of cytochrome c in western blot | | |
| Mouse anti-COX IV  (S2050, BD, 1:500) | | Anti-rabbit IgG, HRP-linked Antibody (#7074, Cell Signaling, 1:5000) | | Detection of COX IV in western blot | | |
| Rabbit anti-Caspase-12 antibody (#ab18766, Abcam, 1:1000) | | Anti-rabbit IgG, HRP-linked Antibody (#7074, Cell Signaling, 1:5000) | | Detection of caspase-12 in western blot | | |
| Rabbit-anti-GRP78 BiP antibody  (#ab21685, Abcam, 1:1000) | | Anti-rabbit IgG, HRP-linked Antibody (#7074, Cell Signaling, 1:5000) | | Detection of GRP78 BiP in western blot | | |
| Rabbit-Phospho-eIF2α antibody (#9721, Cell Signaling, 1:1000) | | Anti-rabbit IgG, HRP-linked Antibody (#7074, Cell signaling, 1:5000) | | Detection of Phospho-eIF2α in western blot | | |
| Rabbit-anti-eIF2α antibody  (#9722, Cell Signaling, 1:1000) | | Anti-rabbit IgG, HRP-linked Antibody (#7074, Cell signaling, 1:5000) | | Detection of eIF2α in western blot | | |
| Rabbit-Phospho SAPK/JNK antibody (#9251, Cell Signaling, 1:1000) | | Anti-rabbit IgG, HRP-linked Antibody (#7074, Cell signaling, 1:5000) | | Detection of Phospho- JNK in western blot | | |
| Rabbit-JNK SAPK/JNK antibody (#9252, Cell Signaling, 1:1000) | | Anti-rabbit IgG, HRP-linked Antibody (#7074, Cell signaling, 1:5000) | | Detection of JNK in western blot | | |
| Mouse-Pan actin antibody  (#MA5-11869, Thermo Fisher Scientific, 1:5000) | | Goat anti-Mouse IgG (H+L) Secondary Antibody, HRP conjugate (#31430, Thermo Fisher Scientific, 1:5000) | | Control for in western blot | | |
| Mouse-anti-CHOP antibody  (#5554, Cell Signaling, 1:100) | | Goat anti-Mouse IgG (H+L) Secondary Antibody, Alexa Fluor® 488 conjugate | | Detection of CHOP in immunofluorescence | | |
| Rabbit- α Tubulin Antibody (H-300)  (#sc-5546, Santa Cruz, 1:1000) | | Goat anti-Rabbit IgG (H+L) Secondary Antibody, Alexa Fluor® 594 conjugate | | Detection of CHOP in immunofluorescence | | |
| Rabbit-NF-κB p65-antibody  (#8242, Cell signaling technology, 1:1000) | | Anti-rabbit IgG, HRP-linked Antibody (#7074, Cell signaling, 1:5000) | | Detection of NF-κB in western blot | | |
| Rabbit-phospho-NF-κB p65 (Ser536) (93H1) antibody  (#3033, Cell signaling technology, 1:1000) | | Anti-rabbit IgG, HRP-linked Antibody (#7074, Cell signaling, 1:5000) | | Detection of phospho-NF-κB in western blot | | |
| Rabbit-IκB-α Antibody  (#9242, Cell signaling technology, 1:1000) | | Anti-rabbit IgG, HRP-linked Antibody (#7074, Cell signaling, 1:5000) | | Detection of total IκB-α  in western blot | | |
| Rabbit-Phospho-IκBα (Ser32) Antibody  (#2859, Cell signaling technology, 1:1000) | | Anti-rabbit IgG, HRP-linked Antibody (#7074, Cell signaling, 1:5000) | | Detection of phospho-IκB-α in western blot | | |
| Mouse-anti-SREBP1 antibody  (#ab3259, Abcam, 1:200) | | Goat anti-Mouse IgG (H+L) Secondary Antibody, HRP conjugate (#31430, Thermo Fisher Scientific, 1:5000) | | Detection of SREBP1 in western blot | | |
| Rabbit -anti-AMPK alpha (phospho) (#ab133448, Abcam1:1000) | | Anti-rabbit IgG, HRP-linked Antibody (#7074, Cell signaling, 1:5000) | | Detection of phospho- AMPK in western blot | | |
| Rabbit -anti-AMPK alpha antibody (#ab3759, Abcam, 1:1000) | | Anti-rabbit IgG, HRP-linked Antibody (#7074, Cell signaling, 1:5000) | | Detection of AMPK in western blot | | |
| Rabbit -anti-ACC antibody (phosphoS221), #ab45174, Abcam, 1:2000 | | Anti-rabbit IgG, HRP-linked Antibody (#7074, Cell signaling, 1:5000) | | Detection of phospho- ACC in western blot | | |
| Rabbit-anti-ACC antibody, #ab45174, Abcam, 1:2000 | | Anti-rabbit IgG, HRP-linked Antibody (#7074, Cell signaling, 1:5000) | | Detection of ACC in western blot | | |
| Rabbit -anti-Cleaved PARP antibody  (#9541, Cell signaling technology, 1:1000) | | Anti-rabbit IgG, HRP-linked Antibody (#7074, Cell signaling, 1:5000) | | Detection of Cleaved PARP in western blot | | |
| Mouse-anti-SREBP2 antibody  (#sc-13552, Santa Cruz, 1:1000) | | Goat anti-Mouse IgG (H+L) Secondary Antibody, HRP conjugate (#31430, Thermo Fisher Scientific, 1:5000) | | Detection of SREBP2 in western blot | | |
| Mouse-anti-PPAR-γ antibody  (#sc-7273, Santa Cruz, 1:1000) | | Goat anti-Mouse IgG (H+L) Secondary Antibody, HRP conjugate (#31430, Thermo Fisher Scientific, 1:5000) | | Detection of PPAR-γ in western blot | | |
| rabbit-anti-PPAR-α antibody  (#sc-9000, Santa Cruz, 1:1000) | | Goat-anti-rabbit IgG, HRP-linked Antibody (#7074, Cell signaling, 1:5000) | | Detection of PPAR-α in western blot | | |
| rabbit-anti-RXR-α antibody  (#sc-774, Santa Cruz, 1:1000) | | Goat-anti-rabbit IgG, HRP-linked Antibody (#7074, Cell signaling, 1:5000) | | Detection of RXR-α in western blot | | |
| Rabbit-anti-cleaved Caspase-3 (Asp175) Antibody  (#CST-9661, Cell signaling technology, 1:100) | | Goat-anti-rabbit-Alexa-594-conjugated Antibody | | Detection of CHOP in immunofluorescence | | |
| **Table S3.** Sequence of the primers used for Q-PCR analysis | | | | | | |
| **Gene (No.)** | | **Primer sequencing (forward and reverse)** | | **Product size** | | **Annealing temperature** |
| *Cd 36* (NM_031561.2) | | 5′-TGG GAA AGT TAT TGC GAC ATG A-3′  5′-TGG GAA AGT TAT TGC GAC ATG A-3′ | | 100 bp | | 59 °C |
| *Vldlr*  (NM_013155.2) | | 5’-GCG GTG CCC ACG AGT TC-3’  5’- GCT CAA GAG ACT CGT CTG ATT GG-3’ | | 105 bp | | 59 °C |
| *Ppargc1a* (NM_031347.1) | | 5'-GAG CGC CGT GTG ATT TAC GT-3'  5'-CGG TGC ATT CCT CAA TTT CA-3 | | 100 bp | | 59 °C |
| *Ppargc1b* (NM_176075.2) | | 5'-TCG GTG AAG GTC GTG TGG TAT AC-3'  5'-GCA CTC GAC TAT CTC ACC AAA CA-3' | | 101 bp | | 59 °C |
| *Pparg*(NM_031347.1) | | 5'-GCT GCA GGC CCT GGA ACT-3'  5'-TGA CAA TCT GCC TGA GGT CTG T-3’ | | 100 bp | | 59 °C |
| *Dgat1* (NM_053437.1) | | 5′-GTC TTT TGG GCG TCA GCT TT-3′  5′-GTG GGA CCT GAG CCA TCA TT-3′ | | 100 bp | | 59 °C |
| *Dgat2 (NM_001012345.1)* | | 5′-GTG GCC TGC AGT GTC ATC CT-3′  5′-TGG GCG TGT TCC AGT CAA-3′ | | 100 bp | | 59 °C |
| *Ppara*  *(NM_013196.1)* | | 5′-AGG AGG CAG AGG TCC GAT TC-3′  5′-TTT GCA AAG CCT GGG ATA GC-3′ | | 100 bp | | 59 °C |
| *Cpt1a*  (NM_031559.2) | | 5′-CCT GAG CAG CGC CAG TCT-3′  5′-GGG ACT CGT CCG GCA CTT-3′ | | 100 bp | | 59 °C |
| *Acox1*  (NM_017340.2) | | 5′-CTC AAG GAG AGT GCT ACG GGT TA-3′  5′-GGA CCG ATA TCC CCG ACA GT-3′ | | 100 bp | | 59 °C |
| *Apoc2*  (NM_001085352.1) | | 5′-GCT CTC CTA GTG TTG GGA AAC G-3′  5′-CCA GTA ACT GAA CAA GTG CTC CTG TA-3′ | | 102 bp | | 59 °C |
| *Lsr*  (NM_032616.1) | | 5′-GCC CAA GAT CTG GAT GGA AA-3′  5′-GCC CCG CCC GAA AAC-3′ | | 100 bp | | 59 °C |
| *Nos2*  (NM_012611.3) | | 5′-GAA AGC GGT CTT TGC TTC T-3′  5′-CGC TTC CGA CTT TCC TGT CT-3′ | | 100 bp | | 59 °C |
| *Tnfa*  (NM_012675.3) | | 5′-GAA AGC GGT CTT TGC TTC T-3′  5′-CGC TTC CGA CTT TCC TGT CT-3′ | | 102 bp | | 59 °C |
| *Il6*  (NM_012589.2) | | 5′-TGA AAC CCT AGT TCA TAT CTT CAA ACA-3′  5′-CCA CTC CTT CTG TGA CTC TAA CTT CTC-3′ | | 100 bp | | 59 °C |
| *Il1b*  (NM_031512.2) | | 5′-TCT GAC CCA TGT GAG CTG AAA G-3′  5′-CGT TGC TTG TCT CTC CTT GTA CA-3′ | | 100 bp | | 59 °C |
| *Hspa5*  (NM_013083.1) | | 5′-GGGACAGGAAACAAAAACAAAATC-3′  5′-TCTCGGCGTCATTGACCAT-3′ | | 100 bp | | 59 °C |
| *Xbp-1*  (NM_001004210.2) | | 5'-TCC GCA GCA CTC AGA CTA CGT-3'  5'-GAA GAG GCA ACA GCG TCA GAA-3' | | 101 bp | | 59 °C |
| *Ddit3*  (NM_001109986.1) | | 5'- GGT CAC AAG CAC CTC CCA AA-3’  3’-TCC GTT TCC TAG TTC TTC CTT GA-‘ | | 101 bp | | 59 °C |
| β-actin  (NM_031144.3) | | 5'-GGC ACC ACA CCT TCT ACA ATG A-3'  5'-ATC TTT TCA CGG TTG GCC TTA G-3' | | 100 bp | | 59 °C |
