# Supplementary material for: Animal Evidence for Synergistic Induction of Hepatic Injury by Dietary Fat and Alcohol Consumption and Its Potential Mechanisms
Source: J Pers Med. 2021 Apr 8;11(4):287. doi: 10.3390/jpm11040287 (PMC8070044; doi:10.3390/jpm11040287)
Supplement: Supplementary file 1 [file jpm-11-00287-s001.zip › Supplementary figure legends.docx]

**Supplementary figure legends**

**Fig. S1.** Changes in body and liver weights and food intake.

Rats were subjected to oral administration of the different doses of ethanol or/and fat diet for 8 weeks. Liver color with the naked eye (A), change of body weight (B), the total gain of body weight (C), and the average of weekly diet intake (D) are presented. Data are expressed as the mean ± SD (*n* = 6). ^†^*p* < 0.05 and ^††^*p* < 0.01 compared with the control group; ^#^*p*< 0.05 compared with the fat diet 40 group; ^**^*p* < 0.01 compared with the ethanol 20 group.

**Fig. S2**. Hepatic parameters for oxidative stress and antioxidant.

Rats were subjected to oral administration of the different doses of ethanol or/and fat diet for 8 weeks. After removal of haptic tissues, parameters for oxidative stress (A to D) and antioxidant (E to H) were measured. Data are expressed as the mean ± SD (*n* = 6). ^†^*p* < 0.05 and ^††^*p* < 0.01 compared with the control group; ^#^*p*< 0.05 compared with the fat diet 40 group; ^*^*p*< 0.05 compared with the ethanol 20 group.

**Fig. S3** Alteration of Capspase-12 activity in HepG2 cells

HepG2 cells were supplemented with PBS, FFA 1 mM, ethanol 2%, or FFA 0.5 mM with ethanol 1% for 24 h. Western blotting analyses for pro caspase-12 and cleaved caspase-12 were conducted.
