# Supplementary figures and images for: Animal Evidence for Synergistic Induction of Hepatic Injury by Dietary Fat and Alcohol Consumption and Its Potential Mechanisms
Source: J Pers Med. 2021 Apr 8;11(4):287. doi: 10.3390/jpm11040287 (PMC8070044; doi:10.3390/jpm11040287)

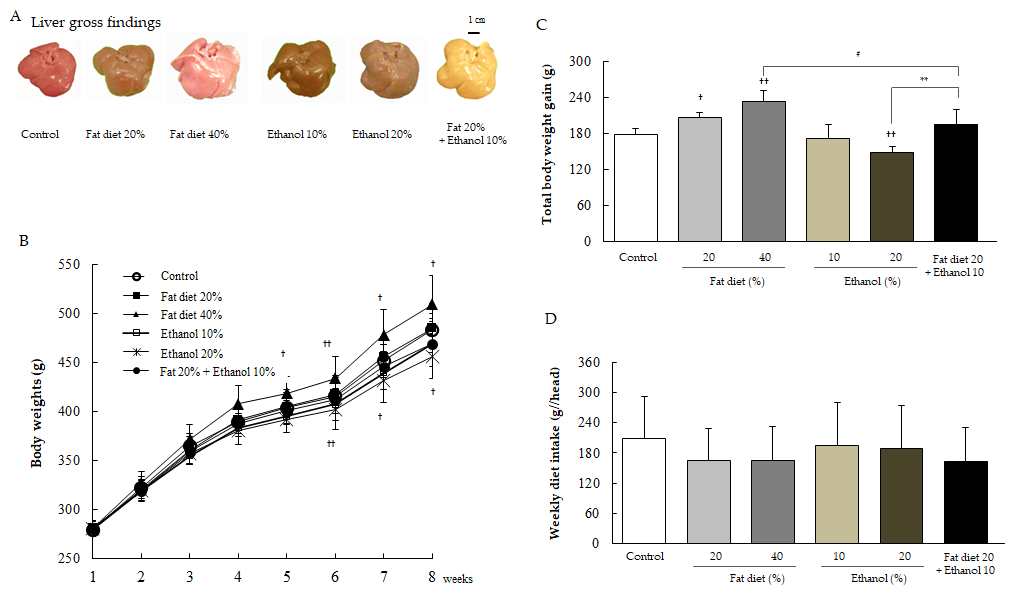

Supplement: Supplementary file 1 [file jpm-11-00287-s001.zip › Fig S1.tif]

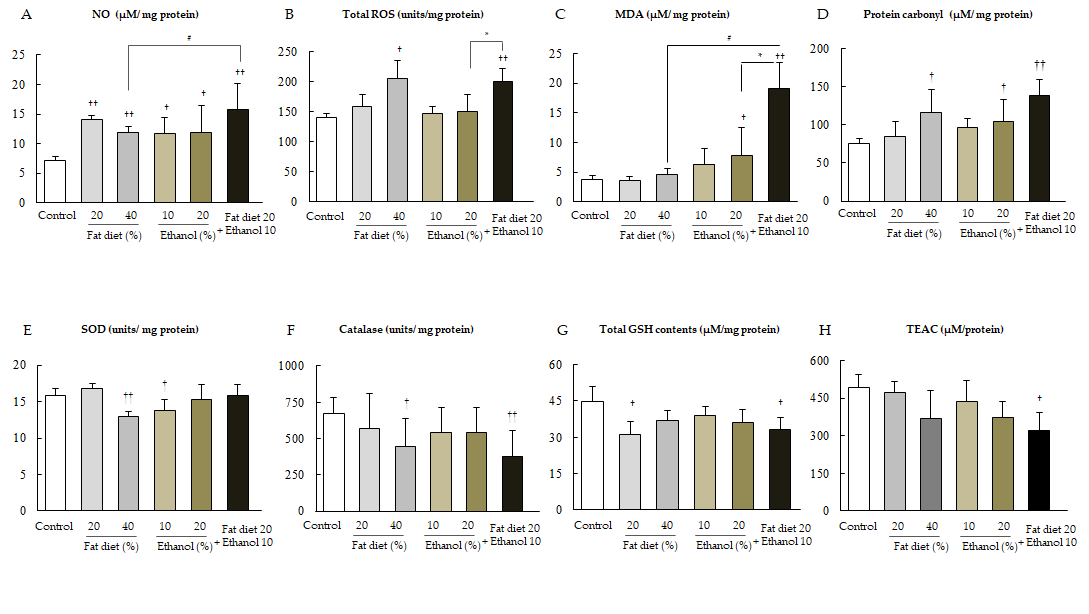

Supplement: Supplementary file 1 [file jpm-11-00287-s001.zip › Fig S2.tif]

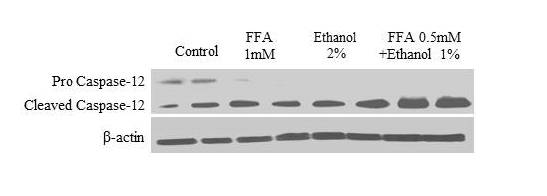

Supplement: Supplementary file 1 [file jpm-11-00287-s001.zip › Fig S3.tif]
